# Multifunctional nanozyme-reinforced copper-coordination polymer nanoparticles for drug-resistance bacteria extinction and diabetic wound healing

**DOI:** 10.1186/s40824-023-00429-z

**Published:** 2023-09-18

**Authors:** Jiahui Zhao, Tengfei Xu, Jichao Sun, Haitao Yuan, Mengyun Hou, Zhijie Li, Jigang Wang, Zhen Liang

**Affiliations:** 1 Department of Geriatrics and Shenzhen Clinical Research Centre for Geriatrics, Shenzhen People’s Hospital (The Second Clinical Medical College, Jinan University, The First Affiliated Hospital, Southern University of Science and Technology), Shenzhen, Guangdong 518020 P. R. China; 2 Integrated Chinese and Western Medicine Postdoctoral Research Station, Jinan University, Guangzhou, 510632 P. R. China; 3 College of Pharmaceutical Sciences, Zhejiang University, Hangzhou, 310058 P. R. China; 4 State Key Laboratory for Quality Ensurance and Sustainable Use of Dao-di Herbs, Artemisinin Research Center, and Institute of Chinese Materia Medica, China Academy of Chinese Medical Sciences, Beijing, China

**Keywords:** Copper-coordination polymer nanoparticles, Reactive oxygen species, Nanozyme, Anti-inflammation, Anti-bacteria

## Abstract

**Background:**

Drug-resistant bacterial infections in chronic wounds are a persistent issue, as they are resistant to antibiotics and can cause excessive inflammation due to generation of reactive oxygen species (ROS). An effective solution would be to not only combat bacterial infections but also scavenge ROS to relieve inflammation at the wound site. Scaffolds with antioxidant properties are attractive for their ability to scavenge ROS, and there is medical demand in developing antioxidant enzyme-mimicking nanomaterials for wound healing.

**Methods:**

In this study, we fabricated copper-coordination polymer nanoparticles (Cu-CPNs) through a self-assembly process. Furthermore, ε-polylysine (EPL), an antibacterial and cationic polymer, was integrated into the Cu-CPNs structure through a simple one-pot self-assembly process without sacrificing the glutathione peroxidase (GPx) and superoxide dismutase (SOD)-mimicking activity of Cu-CPNs.

**Results:**

The resulting Cu-CPNs exhibit excellent antioxidant propertiesin mimicking the activity of glutathione peroxidase and superoxide dismutase and allowing them to effectively scavenge harmful ROS produced in wound sites. The in vitro experiments showed that the resulting Cu-CPNs@EPL complex have superior antioxidant properties and antibacterial effects. Bacterial metabolic analysis revealed that the complex mainly affects the cell membrane integrity and nucleic acid synthesis that leads to bacterial death.

**Conclusions:**

The Cu-CPNs@EPL complex has impressive antioxidant properties and antibacterial effects, making it a promising solution for treating drug-resistant bacterial infections in chronic wounds. The complex’s ability to neutralize multiple ROS and reduce ROS-induced inflammation can help relieve inflammation at the wound site.

**Graphical Abstract:**

Schematic illustration of the ROS scavenging and bacteriostatic function induced by Cu-CPNs@EPL nanozyme in the treatment of MRSA-infected wounds.
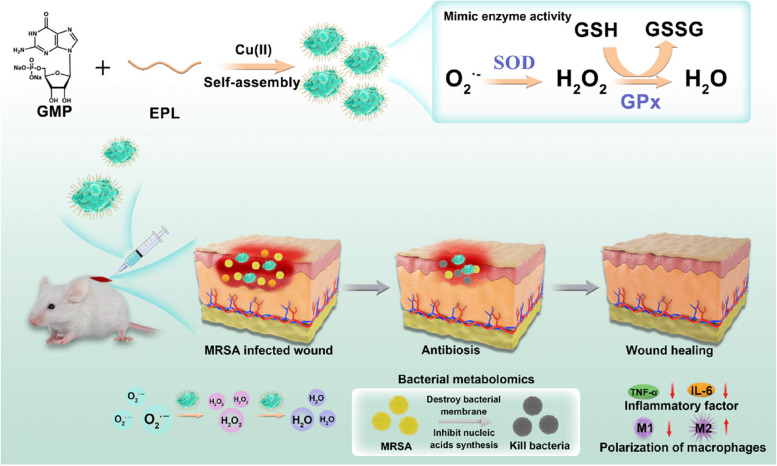

**Supplementary Information:**

The online version contains supplementary material available at 10.1186/s40824-023-00429-z.

## Introduction

Diabetes has high prevalence worldwide and threatens global public health [1, 2]. Diabetic ulceration is a common diabetic complication, and it causes chronic wound infection that is a serious medical problem that threatens the health and quality of life of diabetic patients [3–5]. ROS are generated during incomplete oxygen metabolism, and under diabetic conditions, immune cells increase ROS levels in the wound microenvironment, leading to stubborn scars and prolonged wounds [6–8]. The superfluous ROS within the impaired wound can promote intense inflammatory reactions to make the wounds fragile, but also restricts skin regeneration by stem cells and macrophages [9]. In addition to impeding wound healing, excessive ROS can cause damage to the function of the organism’s macromolecules, leading to oxidative stress. This breaks the redox homeostasis and cause serious harm to the organism. ROS also inhibit vascular regeneration and result in endothelial dysfunction. To combat these negative effects, it is important to develop strategies for scavenging ROS and maintaining redox homeostasis. In addition, diabetic wounds with hyperglycemic microenvironment are very susceptible to recurrent bacterial infections due to hypoimmunity, and the bacterial infection would further elevate the ROS level in wound. In this regard, the process of wound recovery is largely hindered by the abundant oxidative stress in the injured wound [10–12]. To overcome these challenges, it is crucial to develop effective treatments that can simultaneously target bacterial infections and scavenge excessive ROS, promoting the recovery of diabetic wounds.In cellular enzyme-involved metabolism, oxygen undergoes a series of one-electron reactions, which alternately leads to the formation of several kinds of ROS, including superoxide anion (O_2_^•−^), hydroxyl radical (OH·), and hydrogen peroxide (H_2_O_2_). To defend against excessive ROS, the body has endogenous antioxidases, such as catalase (CAT), SOD, and GPx, are capable of catalyzing the disproportionation reactions of O_2_^•−^ and H_2_O_2_ into H_2_O [6, 13, 14]. However, these natural enzymes are usually unstable, with a short half-life in circulation, and are hard to be adequately produced in wound [15]. Nanozymes are promising alternatives to natural enzymes in various applications due to their high stability, low cost, and adjustable catalytic activities [16, 17]. Nanomaterials with enzyme-mimicking activity are promising for regulating cellular redox balance and reducing oxidative damage due to their low cost, large surface area, and stability in harsh conditions. A diversity of nanomaterials, including ceria, manganese dioxide, polydopamine nanoparticles, metal–organic frameworks, and Prussian blue have been developed as efficient nanozymes that mimic antioxidases, providing higher stability and availability compared to endogenous antioxidants, and all of which could potentially alleviate oxidative damage and inflammation reaction in vivo [15, 18–23].

Metalloenzymes are a category of oxidoreductases that initiate cellular redox reactions with biochemicals through using lone pair electrons associated with proton translocation. Nanozymes that mimic single-component oxidoreductases and endogenous antioxidants alone may not effectively alleviate oxidative injury due to the presence of multiple ROS in diabetes. Therefore, it is challenging to formulate nanomaterial with simple structure and remarkable ROS scavenging ability for biomedical applications. Copper ions play a crucial role in cellular redox reactions as redox cofactors for enzymes such as tyrosinase, laccase, and Cu/Zn-SODs. Copper ions cycle between Cu(II) and Cu(I) states in these reactions [24–26]. The Cu(I) form is the important component that travels and senses intracellularly, so it is vital to keep cellular copper in the Cu(I) valence state for optimal biomedical use [27]. Thus, it is essential to develop copper-based nanomaterials with Cu(I) state component which are capable of scavenging endogenous ROS. In addition, it was reported that copper ions also have a strong impact on wound healing through contributions to angiogenesis and collagen deposition [28–31].

**Fig. 1:**
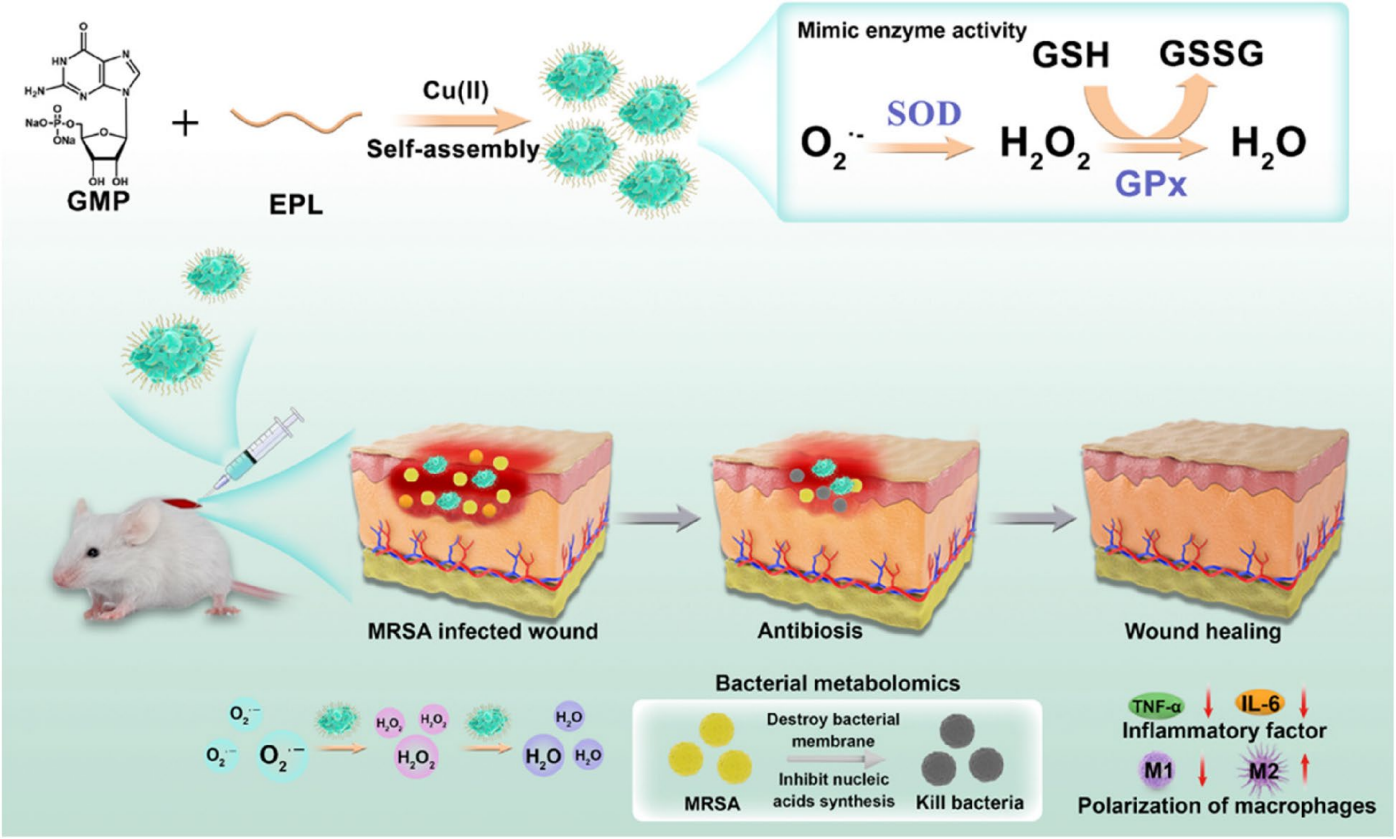
Schematic illustration of the ROS scavenging and bacteriostatic function induced by Cu-CPNs@EPL nanozyme in the treatment of MRSA-infected wounds

The study aims to develop a solution to treat drug-resistant bacterial infections in chronic wounds with the Cu-CPNs complex which was endowed with antioxidant properties to scavenge harmful ROS produced in wound sites and relieve inflammation. We fabricated this antioxidant nanozyme system to reduce the level of ROS and enhance wound healing. The nanozyme was prepared using guanosine monophosphate (GMP) as the coordination scaffold and copper ion as the center of coordination polymer nanoparticles (CPNs) (Fig. 1). The resultant Cu-CPNs@EPL complex exhibited impressive antioxidant properties and antibacterial activity, making it valuable for potential clinical translation into treatments against drug-resistant bacterial infections in chronic wounds.

## Materials and methods

### Materials

Copper chloride dihydrate (CuCl_2_·2H_2_O), glutathione, methionine, 5,5’-dithiobis (2-nitrobenzoic acid) (DTNB), glutaraldehyde solution (25%), EPL, glucose, gelatin, GMP, streptozocin (STZ), citric acid, sodium citrate, 2’,7’-dichlorofluorescein diacetate (DCFH-DA) and cell counting kit-8 (CCK-8) were purchased from Sigma-Aldrich (Shanghai, China). Riboflavin, nitrotetrazolium blue chloride (NBT), *Luria–Bertani* culture (LB) and LB Agar culture were purchased from Sangon Biotech Co., Ltd. (Shanghai, China), Paraformaldehydesolution (PFA, 4%) was purchased from Beyotime Biotechnology (Shanghai, China). Hydrogen peroxide (30.0%), and ethanol (99.7%) were purchased from Sinopharm Chemical ReagentCo., Ltd (Shanghai, China). Syto 9/PI live/dead bacterial double stain kit was purchased from Thermo Fisher Scientific (Waltham, USA). Monocolonies of Methicillin-resistant *Staphylococcus aureus* (MRSA, ATCC43300) and *Pseudomonas aeruginosa* PAO1 (PAO1, CGMCC 1.12483) were purchased from the China Center of Industrial Culture Collection (Beijing, China) and BeNa Culture Collection (Suzhou, China), respectively. Raw 264.7 and NIH 3T3 cells were purchased from the American Type Culture Collection (Manassas, VA, USA).

### Apparatus and characterization

UV/Vis absorption and fluorescence spectra measurements were performed on a multimode reader Spark® 10M (Tecan, Männedorf, Switzerland). Scanning electron microscopy (SEM) images were obtained on a field-emission scanning electron microscope with X-MaxN energy spectrum (ZEISS, Jena, Germany). X-ray photoelectron spectroscopy (XPS) data were recorded using K-Alpha using Al Kα (hv = 1486.6 eV) radiation (Thermo Scientific, Waltham, USA). X-ray diffraction (XRD) patterns were recorded from a D8 ADVANCE (Bruker, Karlsruhe, Germany) X-ray diffracto meter with Cu Kα radiation (λ = 1.5406 Å). Fourier transform infrared spectra were obtained using a Bruker ALPHA spectrophotometer (Bruker, Karlsruhe, Germany). The zeta potential was measured with a Zetasizer Nano ZS DLS system (Malvern Instruments Ltd., Malvern, England). N_2_ adsorption/desorption isotherms were obtained using an ASAP 2020 HD88/Autosorb IQ system (Quantachrome, Florida, USA) at 77 K. Fluorescence microscope images were recorded by NIS-Elements Viewer (Nikon, Tokyo, Japan). Mass spectrum data were collected from QE (Thermo Scientific, Waltham, USA).

### Preparation of Cu-CPNs and Cu-CPNs@EPL

We first design and synthesize copper-nanoparticles with oxidase or reductase-mimicking activity. To address this issue, nucleotides were used as metal ligands due to their ability to coordinate with transition metal ions through various interactions, such as the lone-pair electrons of nitrogen and oxygen atoms in nucleobases and phosphate groups [25]. Inspired by the fact that metal catalytic center served as the cofactor of some natural oxidoreductases, it has been found that copper ion could coordinate with GMP to form amorphous CPNs [16]. To prepare Cu-CPNs, CuCl_2_ aqueous solution (20 mM, 5 mL) was added to the GMP aqueous solution (15 mM, 5 mL), and the mixture was placed on magnetic stirring at 37℃ for 2 h. Afterwards, the produced wathet blue turbid solution was centrifuged for 10 min at 8500 rpm, and the precipitate was collected after being washed with water twice and re-dispersed in 10 mL of water for further use. To prepare Cu-CPNs@EPL, the procedure was similar to that of Cu-CPNs: CuCl_2_ aqueous solution (20 mM, 5 mL) was added to 5 mL mixture solution of EPL (5 mg/mL) and GMP (15 mM), and the mixture was placed on magnetic stirring at 37 ℃ for 2 h. The obtained turbid solution was centrifuged at 8500 rpm for 10 min, and the blue precipitate was collected after being washed with water twice and re-dispersed in 10 mL of water for further use.

### The SOD-like activity of Cu-CPNs and Cu-CPNs@EPL in scavenging of O_2_^•−^

**Fig. 2:**
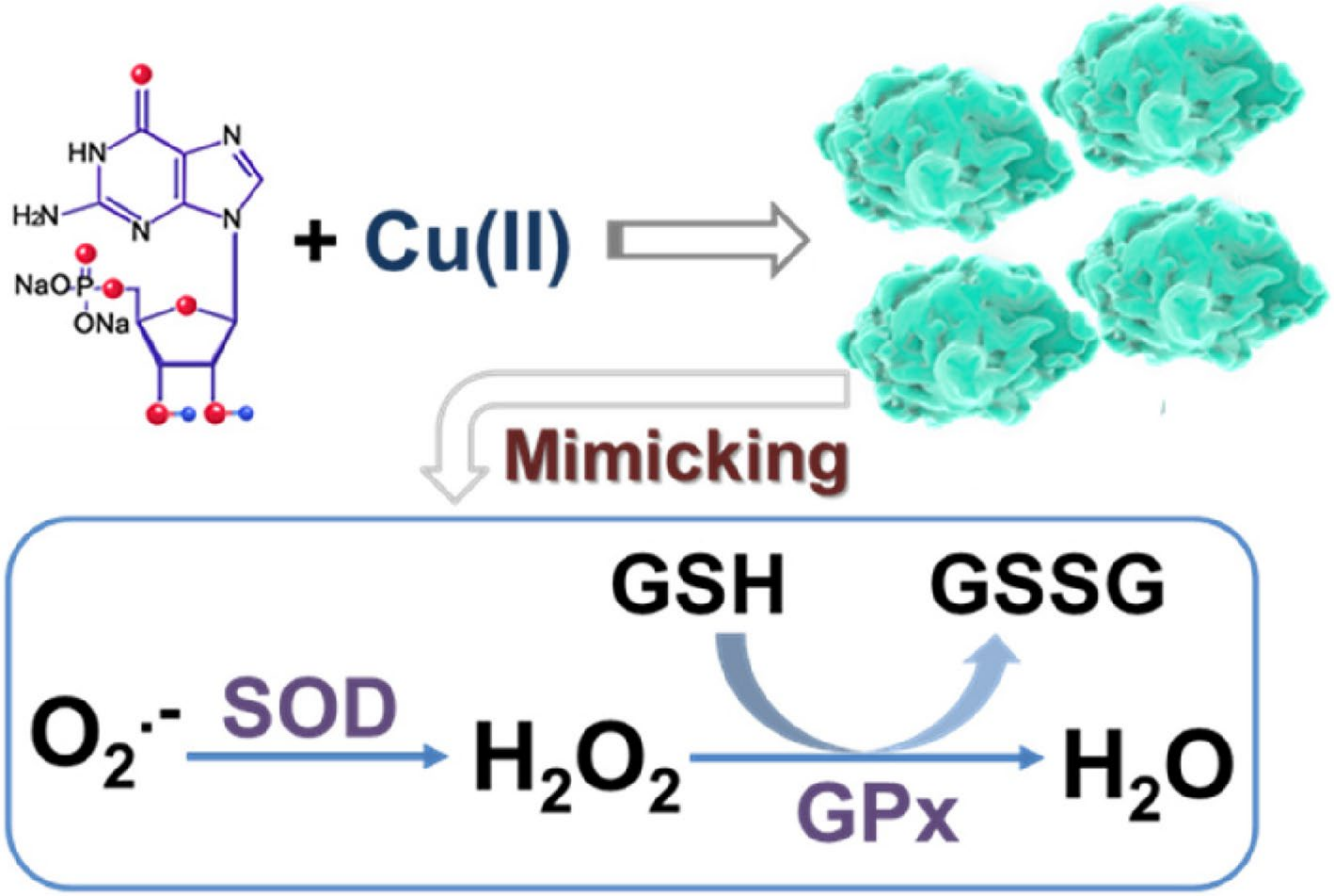
Schematic representation of Cu-CPNs as a mimic of antioxidant nanozymes, (typically, SOD and GPx) in scavenging ROS

This method measures the scavenging efficiency of O_2_^•−^ by examining the inhibition of formazan formation. A solution of NBT and riboflavin was illuminated with 30 W bright light for 2 min, with varying concentrations of Cu-CPNs added. Under illumination, the reaction between riboflavin and oxygen produced O_2_^•−^, aided by photo-excited reduction of riboflavin. O_2_^•−^ then reduced yellow NBT to blue formazan, but the reaction of Cu-CPNs with O_2_^•−^ produced O_2_ and H_2_O_2_, which inhibited formazan formation [32]. This indicated the SOD-mimicking activity of the prepared Cu-CPNs, as shown in Fig. 2. The intensity of the blue color of the reaction solution after photoreduction is inversely proportional to the SOD-like activity; a darker blue color indicates lower activity, while a lighter blue color indicates higher activity. Here, the SOD activity assay solutions containing riboflavin (20 μM), methionine (10 mM), NBT (100 μM) were prepared in PBS (10 mM, pH 7.4). Then different concentrations of Cu-CPNs and Cu-CPNs@EPL solutions (0–200 μg/mL) were added to the assay solutions, respectively. And the mixtures were illuminated upon white light for 5 min. After illumination, the absorbance spectra of mixtures were measured. Sample containing riboflavin, methionine, and NBT after illumination was defined as positive control.

### The GPx-like activity of Cu-CPNs and Cu-CPNs@EPL

The GPx-like activity was estimated through the GSH determination using Ellman reagent (DTNB solution) [18]. Typically, the GPx activity assay solutions were consisted of GSH (1 mM), H_2_O_2_ (1 mM) and 20 μg/mL Cu-CPNs or 20 μg/mL Cu-CPNs@EPL in PBS (10 mM, pH 7.4). After incubation at room temperature for 30 min, DTNB solution (1 mM) was added into the assay solutions, the absorbance spectra of mixtures were measured in 15 min. Sample containing GSH (1 mM) and 20 μg/mL Cu-CPNs or 20 μg/mL Cu-CPNs@EPL was defined as negative control without H_2_O_2_, and sample containing GSH (1 mM) and H_2_O_2_ (1 mM) was defined as negative control without Cu-CPNs or Cu-CPNs@EPL.

### Intracellular ROS (H_2_O_2_) depletion assay by Cu-CPNs and Cu-CPNs@EPL

To assess their cytoprotective properties, the effects of Cu-CPNs and Cu-CPNs@EPL on ROS damage were investigated, using the ROS indicator DCFH-DA for intracellular ROS imaging and quantification [33]. The intracellular ROS-scavenging ability of Cu-CPNs and Cu-CPNs@EPL was tested using Raw 264.7 and NIH 3T3 cells. The cells were cultured in Dulbecco’s modified Eagle’s medium (DMEM) supplemented with 10% fetal bovine serum at 37 °C in an incubator supplied with an atmosphere of 5% CO_2_. To investigate the intracellular ROS scavenging ability, Raw 264.7 and NIH 3T3 cells were seeded into 24-well plates at the density of 10 × 10^4^ cells per well, respectively. After 24 h incubation, 10 μg/mL Cu-CPNs or Cu-CPNs@EPL was added to each group of wells, respectively. After incubation for 30 min, the cells were treated with 200 μM H_2_O_2_ and further incubated at 37 °C for 24 h. Wells without the addition of H_2_O_2_ and Cu-CPNs were regarded as the negative control, and wells without the addition of Cu-CPNs were regarded as the postive control. After washing the cells with PBS, 10 μM DCFH-DA in serum-free DMEM was added to the harvested cells, which were then incubated at 37°C for 20 min. The cells were washed three times with serum-free DMEM to remove any remaining DCFH-DA, and DMEM was added to the cells for observation under a fluorescence microscope using an excitation wavelength of 488 nm.

### Cell culture and cytotoxicity assay

For visualization of cell proliferation in vitro, Raw 264.7 and NIH 3T3 cells (5 × 10^3^/well) were seeded in a 48-well plate. The as-prepared Cu-CPNs and Cu-CPNs@EPL were incubated with cells for 24 h. Cell activity was assessed using a Live/Dead cell staining kit. Live cells were detected by green fluorescence produced from Calcein (Ex/Em = 494/517 nm), while dead cells were detected by red fluorescence with Propidium iodide (PI, Ex/Em = 535/617 nm). The staining procedures were conducted according to the manufacturer’s protocol. The cells were observed under a fluorescence microscope using an excitation wavelength of 505 nm.

Futhermore, Raw 264.7 and NIH 3T3 cell lines were seeded in 96-well plates and incubated with varying concentrations of Cu-CPNs (0, 3.12, 6.25, 12.5, 25, 50, 100 μg/mL) or Cu-CPNs@EPL (0, 3.12, 6.25, 12.5, 25, 50, 100 μg/mL) for 24 h. After incubation, cell viability was assessed using the CCK-8 assay, which measures absorbance intensity at 450 nm.

### In vitro antibacterial experiments

Monocolonies of MRSA and PAO1 were transferred to LB broth and shaken at 200 rpm and 37 °C for 3 h. The bacteria were then diluted in LB broth to 1 × 10^7^ CFU/mL. For the in vitro antibacterial assay, four groups of as-prepared bacterial suspensions (200 µL, 1 × 10^7^ CFU/mL). The growth-inhibition investigation was performed in a liquid LB medium: four groups of as-prepared bacterial suspensions were treated with I) PBS, II) Cu-CPNs, III) EPL, IV) Cu-CPNs@EPL in 10 mM phosphate buffer, and incubated at 37 °C and 200 rpm/min for 6 h. Then the bacterial concentrations were evaluated by monitoring the optical density at 600 nm (OD_600_).

### Morphology study and live/dead staining of bacterial cells

The SEM characterization was used to monitor the changes of the bacteria morphology. The bacterial suspension was treated the same as in the antibacterial experiments.The obtained bacterial suspension was washed three times with PBS and collected by centrifugation at 8500 rpm for 3 min, followed by preservation in 4% glutaraldehyde at room temperature for 0.5 h in darkness. After that, the bacteria were dehydrated in a series of gradient concentrations (30–100%) of ethanol solutions for 5 min. Finally, the bacterial samples were dried by nitrogen gas flow and coated with gold by sputtering and then observed by a SEM.

### Metabolite extraction of Cu-CPNs treated bacteria

The MRSA bacteria were treated with PBS, Cu-CPNs, EPL and Cu-CPNs@EPL in LB broth and shaken at 200 rpm and 37 °C, respectively. After 6 h treatment, the bacteria were washed with ice-cold PBS twice and quenched with pre-cold methanol: water (1:1, -40 °C), sonicated in ice bath for 5 min, followed with freeze-thaw for three times, and then centrifugated at 20,000 g for 10 min at 4 °C to obtain the supernatant and precipitation. The supernatant was dried in a freeze dryer for subsequent liquid chromatography-tandem mass spectrometry (LC-MS/MS) analysis, while the precipitated protein was measured for the normalization of the resuspended volume.

### Metabolomics data acquisition

The dried supernatant was resuspended with ice-cold acetonitrile: water (1: 1), vortexed for 30 s, and centrifugated at 20,000 g for 10 min at 4 °C for the subsequent LC-MS/MS analysis. The metabolite separation was performed on a Waters ACQUITY UPLC BEH Amide column (particle size, 1.7 µm; 100 mm (length) × 2.1 mm (i.d.)) in the negative ion mode, with the mobile phase A (100% H_2_O + 25 mM CH_3_COONH_4_ + 25 mM NH_4_OH) and B (acetonitrile), with the gradient elution conditions set as 0–1 min, B keeps at 95%; 1–14 min, B decreases to 65%; 14–16 min, B decreases to 40% and then kept to 18 min; 18.1–23 min, B keeps at 95%. The column temperature was kept at 25 °C, with the flow rate at 0.3 mL/min. In the positive ion mode, the metabolite separation was performed on a Thermo Hyperil Gold C18 column (100 × 2.1 mm, 1.9 μm), with the mobile phases consisting of A: 0.1% formic acid in H_2_O and B: 0.1% formic acid in acetonitrile. The gradient elution was set as follows: 0–1 min, 5% B, 1–12 min, 5%-100% B, 12.1–15 min, 5% B. The MS data acquisition was performed by Q Exactive Plus (ThermoFisher Scientific, Rockford, IL) system, in full scan MS mode with 70,000 resolution. The spray voltage was set as 3.5 and 3.2 kV for positive and negative mode, respectively. The capillary and aux gas heater temperature was set as 320 °C and 350 °C, respectively. The flow rate of the sheath and aux gas was set at 35 and 15 arbitrary units, respectively.

### Metabolomic data processing

The raw LC-MS/MS data were first processed by using Compound Discoverer 3.2 (Thermo Fisher Scientific), and the metabolites were annotated by the databases like mzCloud, mzVault, Masslist and Chemspider. Principal component analysis (PCA) and cluster analysis of the identified metabolites were performed in R (version 3.6.3).

### In vivo diabetic wound healing evaluation

To further investigate its in vivo efficacy, we used a diabetic mouse model. The model was established by administering STZ to C57BL/6 mice, a widely used method for studying chronic wound healing [7]. The effectiveness of Cu-CPNs@EPL in treating diabetic cutaneous wounds infected by MRSA was evaluated in a mouse model. The diabetic condition of mice was confirmed by the elevated mean blood glucose concentrations of 21 ± 2.6 mM at day 0 and 29 ± 2.3 mM at day 12, which were induced by intraperitoneal injection of STZ for five consecutive days.

C57BL/6 male mice were purchased from GemPharmatech (Jiangsu, China). All animal protocols in this study were approved by the Animal Care and Use Committee of Laboratory Animal Center of Shenzhen People’s Hospital (approval number: AUP-211009-WJG-0001-01). The male 8-week old C57 mice were intra-peritonelly injected with streptozotocin (50 mg/kg) for five consecutive days to induce the Type 1 diabetes model according to previous reports [1]. After 2 weeks, blood glucose was measured using a commercial glucometer (Yuwell, China), and mice with a blood glucose level above 16.7 mM were considered as diabetic. The diabetic mice were anesthetized with 2.5% avertin solution, and the hair on the back were shaved off. A round full-thickness cutaneous wound area (8 mm diameter) was created on the back, and then 40 µL of MRSA solution (2.5 × 10^8^ CFU/ml) was introduced onto the wound. The wounds were covered with Tegaderm Film and gauzeto obtain the infected wound model after 24 h of infection. Mice were then randomly assigned into four groups, with six mice in each group. The mice were treated with 40 µL PBS, Cu-CPNs, EPL, Cu-CPNs@EPL in gelatin hydrogel. Wound images were captured using a camera on cellphone (Huawei, China) at day 0, 3, 7, 14, and 21, and the blood glucose was measured by tail blood collection. At the last day of treatment, the mice were sacrificed after anesthesia and blood collection, and the wound tissues and major organs were collected and preserved in 4% paraformaldehyde solution for further analysis.

### Histological analysis

Hematoxylin and eosin (H&E) staining, Masson’s trichrome staining (MTS), and Giemsa staining were performed forhistological analysis according to the manufacturer’s instructions, and slides were then observed under a microscope. The immunostaining of cytokeratin 14 (CK14), CD31, CD86 were performed to evaluate the epithelialization, angiogenesis and pro-inflammatory M1 macrophages after the treatment period, respectively. The immunofluorescence staining of TNF-α and IL-6 were performed to the pro-inflammatory cytokines in the wound tissue.

## Results

### Synthesis and characterization of coordination polymer nanoparticles

**Fig. 3:**
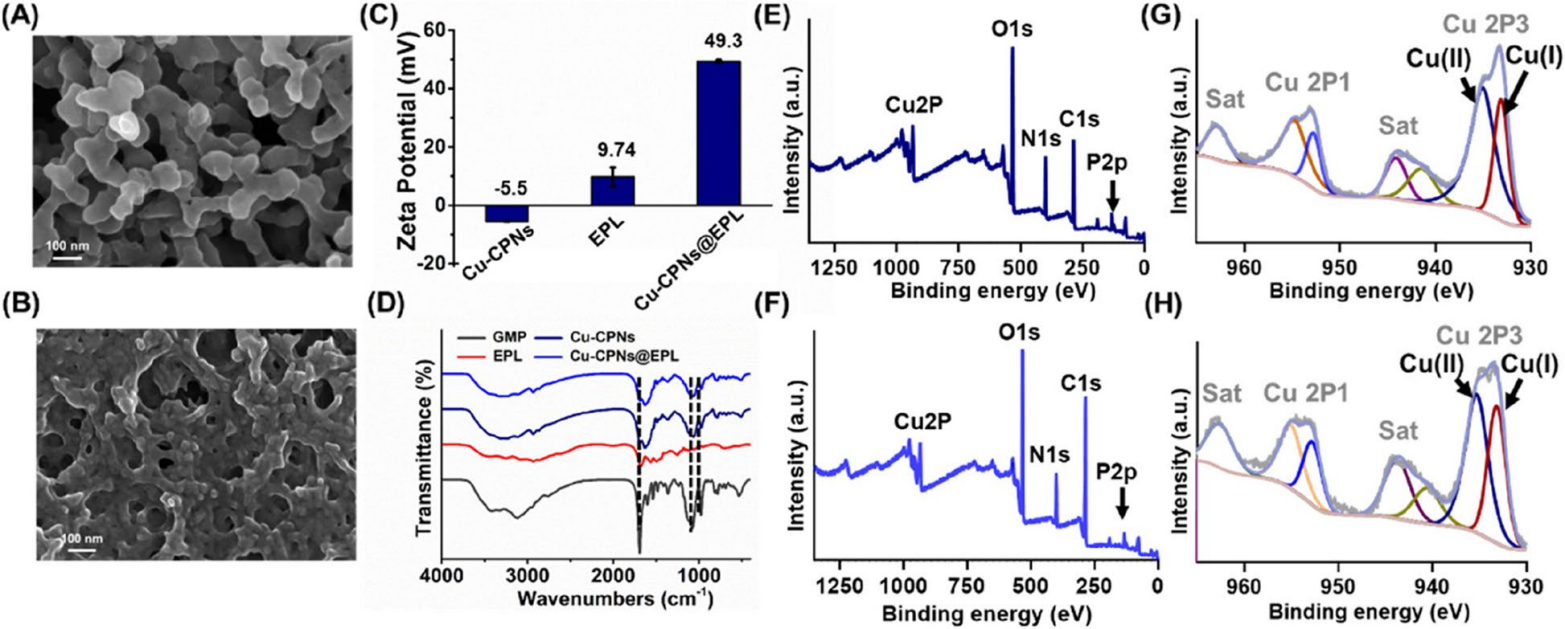
A representative TEM image of **A** Cu-CPNs and **B** Cu-CPNs@EPL; **C** Zeta potential of Cu-CPNs, EPL, and Cu-CPNs@EPL; **D** FTIR spectra of GMP, Cu-CPNs, EPL, and Cu-CPNs@EPL; XPS fully scanned spectra of **E** Cu-CPNs and **F** Cu-CPNs@EPL; Cu 2P XPS peaks of **G** Cu-CPNs and **H** Cu-CPNs@EPL

The structural unit of Cu-CPNs is depicted in Fig. S1. Figure 3A shows the morphological characteristics of the Cu-CPNs, which were characterized using SEM. The Cu-CPNs exhibit a coordination polymer network structure made of interwoven irregular nanoparticles with a diameter of 80–100 nm. The zeta potential of the Cu-CPNs was measured to be -5.5 eV. EPL is an-FDA approved cationic antimicrobial polypeptide with high biocompatibility [34, 35], and it was used to improve the antibacterial properties of the Cu-CPNs. During the self-assembly process, EPL was added to the Cu-CPNs to produce the Cu-CPNs@EPL complex via “one-pot” electrostatic assembly, and the SEM image of the Cu-CPNs@EPL was displayed in Fig. 2B. The zeta potential measurements (Fig. 3C) and SEM analysis confirmed the production of the Cu-CPNs@EPL complex, which showed strong electropositivity and the ability to interact with bacteria for potent antibacterial effects. The FTIR spectra of the Cu-CPNs and Cu-CPNs@EPL were similar to those of GMP and EPL, respectively, with no evident shift in the FTIR peak of GMP (Fig. 3D). The XRD and XPS analyses were used to investigate the physical and chemical properties of the Cu-CPNs and Cu-CPNs@EPL. The XPS full spectrum and core peak were used to determine the element species and copper valence state on the surface of the Cu-CPNs and Cu-CPNs@EPL. Figure 3E and F show that Cu, N, O, P, and C elements coexisted in the Cu-CPNs and Cu-CPNs@EPL, and the Cu 2p signal in Fig. 3G and H verified the coexistence of Cu(II) and Cu(I) states in the Cu-CPNs and Cu-CPNs@EPL [36]. The XRD spectra indicated an amorphous state of the Cu-CPNs and Cu-CPNs@EPL (Fig. S2). The N_2_ adsorption-desorption results in Fig. S3 showed the porous property of the Cu-CPNs, with a surface area of 3.49 m^2^/g and an average pore diameter of 10.14 nm, and the porous Cu-CPNs@EPL had a surface area of 16.26 m^2^/g and an average pore diameter of 16.77 nm.

### SOD and GPx mimetic activity of Cu-CPNs and Cu-CPNs@EPL

**Fig. 4:**
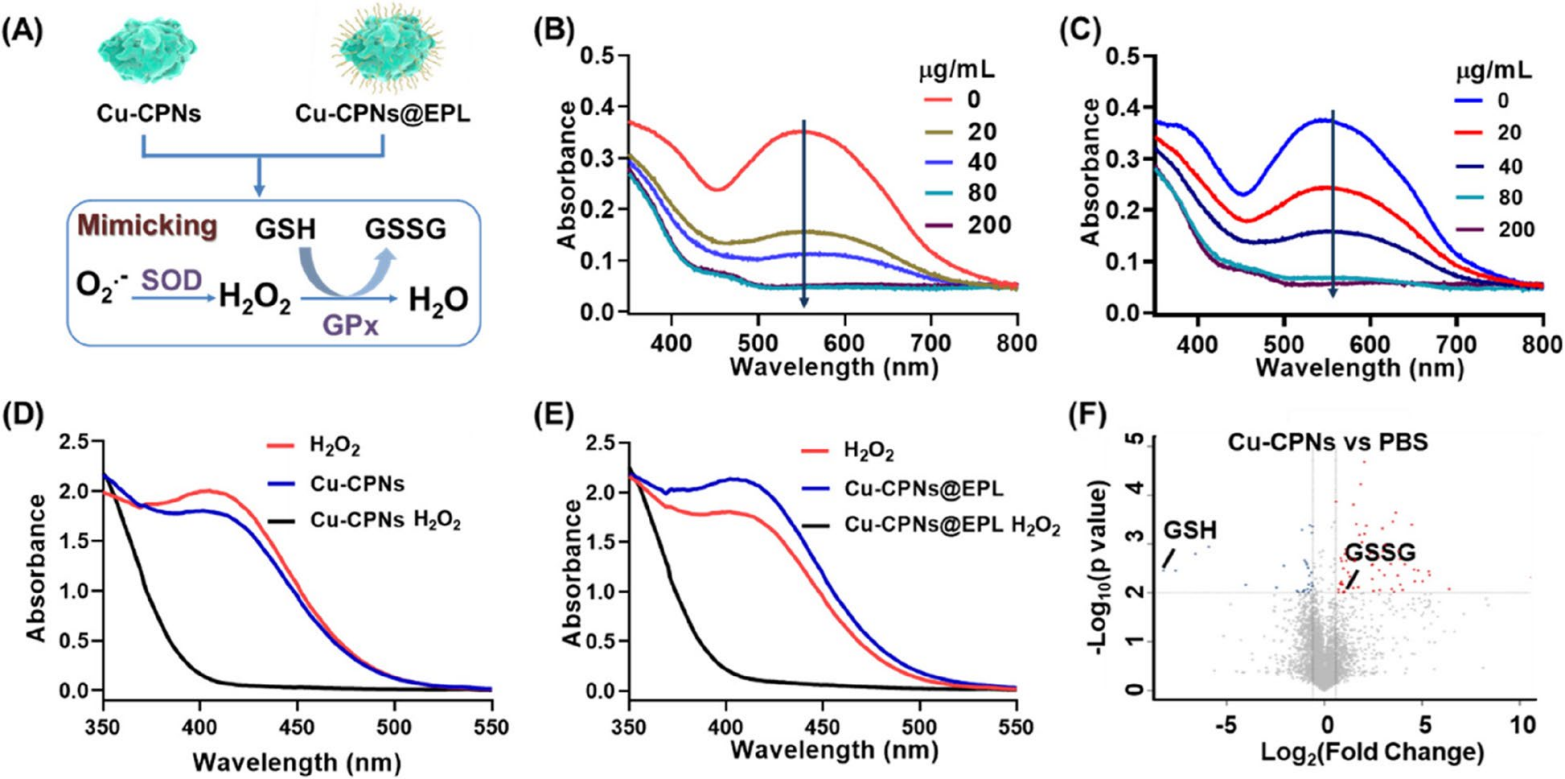
**A** Schematic representation of Cu-CPNs as the antioxidant nanozyme (typically, SOD and GPx) in ROS scavenging; the SOD-mimicking activity of **B** Cu-CPNs and **C** Cu-CPNs@EPL in scavenging efficiencies of O_2_^•−^ using NBT as the indicator; the GPx-mimicking activity of **D** Cu-CPNs and **E** Cu-CPNs@EPL; **F** The volcano plot of the metabolic profiling change in comparison of Cu-CPNs and PBS treated metabolites, the metabolites were extracted from mouse liver tissue

The reduction in UV-vis absorbance peak at 560 nm after the reaction was observed for both Cu-CPNs and Cu-CPNs@EPL, showing their effective SOD-mimicking activity. The SOD-mimicking activity of Cu-CPNs and Cu-CPNs@EPL was measured by the riboflavin-photoreduction of NBT method, which is based on the inhibition of formazan formation. The UV-vis absorbance peak at 560 nm (indicator of formazan) decreased in a concentration-dependent manner for Cu-CPNs (Fig. 4B), indicating its effective SOD-mimicking activity. Similarly, the decreased absorbance peak was also observed for Cu-CPNs@EPL (Fig. 4C).

Furthermore, the GPx-mimicking activity of Cu-CPNs was estimated through the GSH/ oxidized glutathione (GSSG) system by using Ellman reagent (DTNB solution), in which GSH can react with DTNB to produce a characteristic absorption at 412 nm. As shown in Fig. 4D, there was still characteristic absorbance peak at 412 nm when H_2_O_2_ or Cu-CPNs was separately incubated with the assay solution containing GSH and DTNB. However, the typical absorbance peak at 412 nm disappeared when the mixture of H_2_O_2_ and Cu-CPNs with assay solution was oxidized to GSSG (Fig. 4D). The similar result was obtained for Cu-CPNs@EPL as well (Fig. 4E). The results shown in the volcano plot in Fig. 4F, revealed an increase in GSSG after Cu-CPNs treatment, further validating its GPx-mimicking activity.

**Fig. 5:**
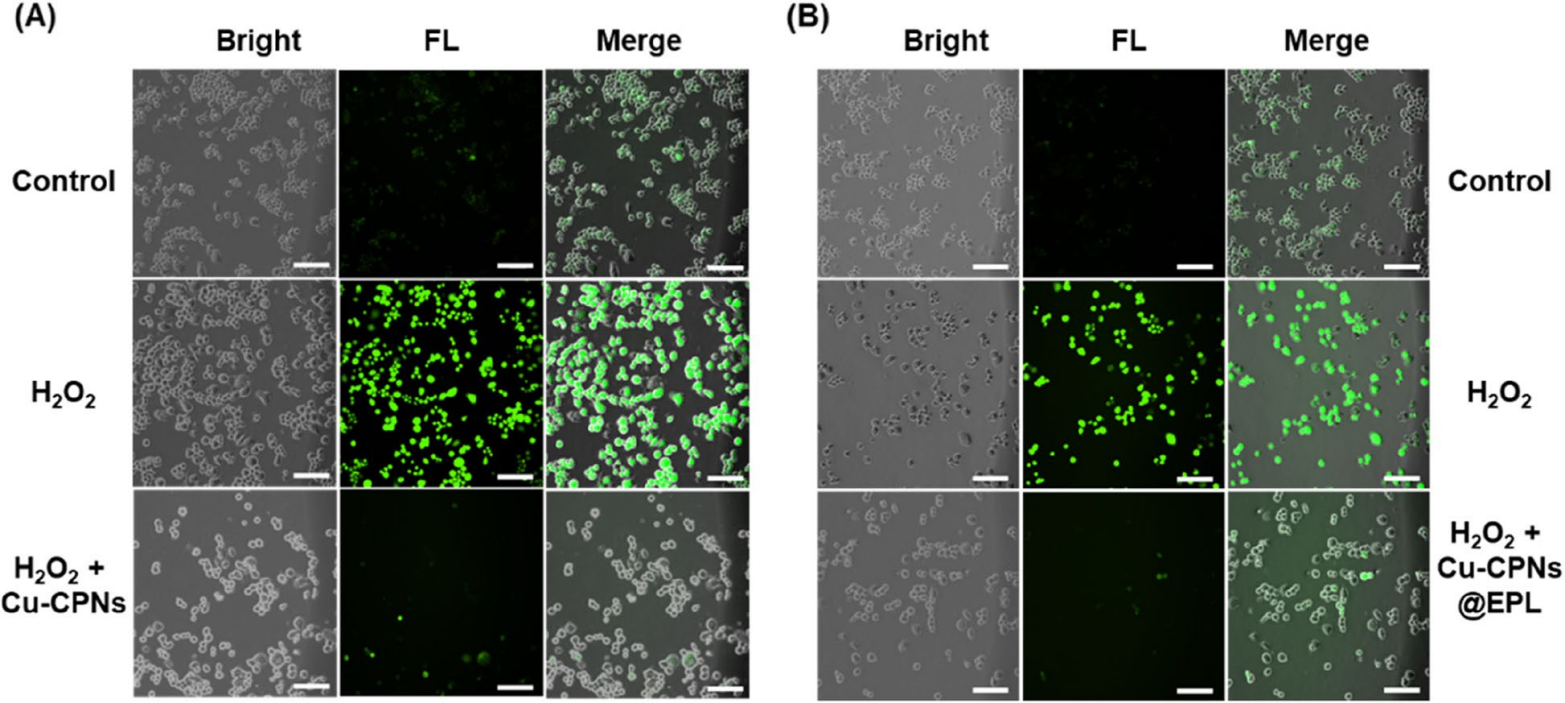
Characteristic picture of ROS staining (green fluorescence) in Raw 264.7 cell under **A** Cu-CPNs and **B** Cu-CPNs@EPL treatment (Scale bar: 100 μm)

The cytotoxicity of Cu-CPNs and Cu-CPNs@EPL was evaluated using CCk-8 and Live/Dead staining assays on Raw 264.7 and NIH 3T3 cells. The results from the CCk-8 assay (Fig. S4A and B) showed that Cu-CPNs and Cu-CPNs@EPL at concentrations of 0–50 μg/mL did not significantly inhibit cell growth. The Live/Dead staining assay (Fig. S4C and D) also showed no significant cell death upon treatment with 25 μg/mL of Cu-CPNs or Cu-CPNs@EPL. The DCFH-DA reacts with cellular ROS to produce a green fluorescent DCF. Raw 264.7 cells showed low intracellular ROS levels that were barely detectable by fluorescence microscopy, as shown in Fig. 5A and B. The ROS level increased dramatically in Raw 264.7 cells after treatment with 200 μM H_2_O_2_ (green fluorescent signal). However, when the cells were pretreated with 20 μg/mL of Cu-CPNs or Cu-CPNs@EPL, the intracellular ROS level significantly decreased, demonstrating the in vitro ROS-scavenging ability of Cu-CPNs or Cu-CPNs@EPL. The ROS-scavenging ability of Cu-CPNs was also tested in another cell line, mouse embryonic fibroblast cell NIH 3T3, and was found to be effective, as shown in Fig. S5.

### In vitro antibacterial properties

**Fig. 6:**
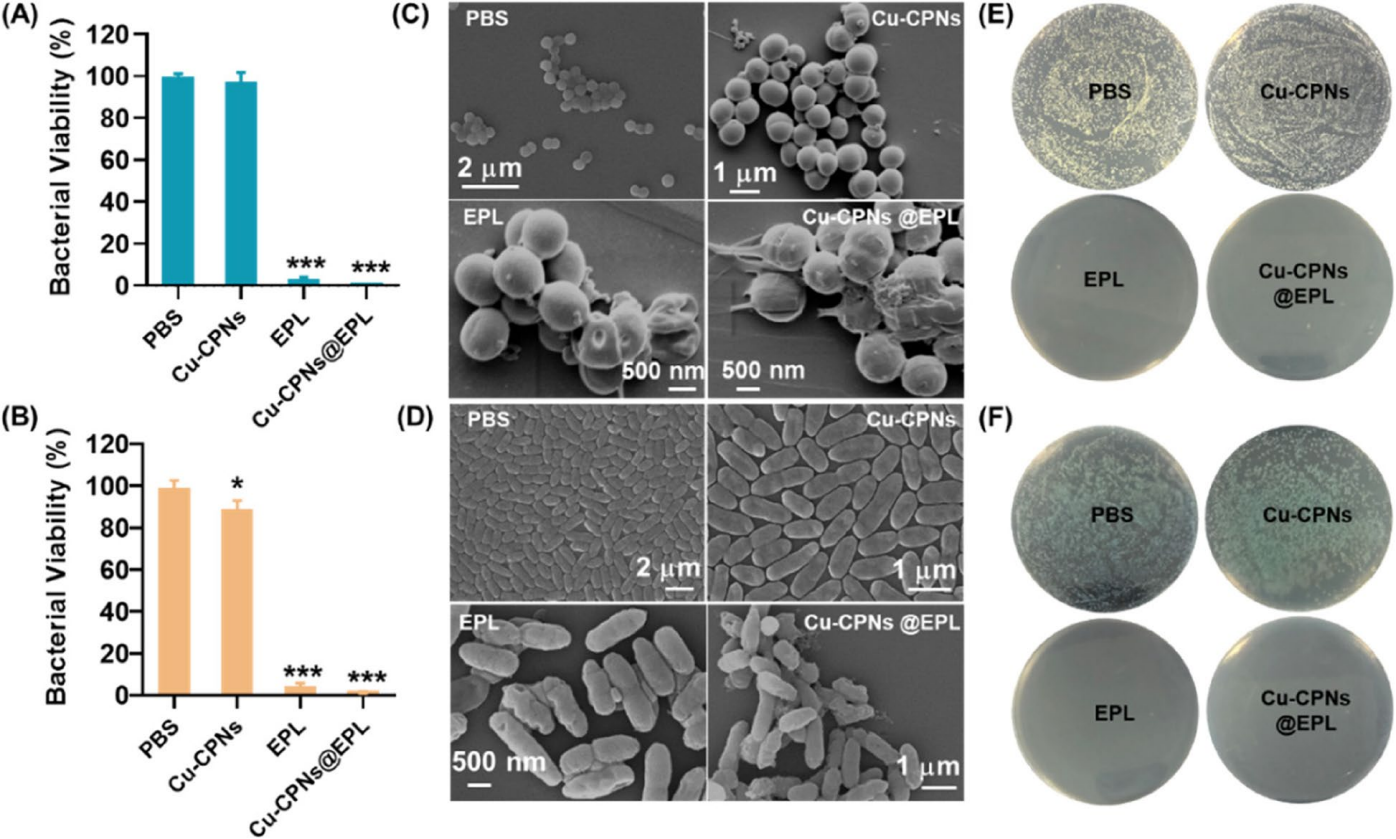
The viability of **A** MRSA and **B** PAO1 after treatment with PBS, 25 μg/mL Cu-CPNs, 25 μg/mL EPL, and 25 μg/mL Cu-CPNs@EPL; significant difference (Bars represent SD, *P < 0.05, ***P < 0.01, with four replicates in each group) compared with PBS group from the collected data; SEM images of **C** MRSA and **D** PAO1 after exposure to PBS, Cu-CPNs, EPL, and Cu-CPNs@EPL; and bacterial colony photographs of **E** MRSA and **F** PAO1 after corresponding treatment

The results in Fig. 5A and B show that the Cu-CPNs@EPL, with its strong electropositive property, effectively adheres to the negatively charged bacterial membrane and demonstrates broad-spectrum antibacterial efficiency against MRSA and PAO1 when incubated at a concentration of 2 × 10^7^ CFU/mL. Results showed that EPL and Cu-CPNs@EPL had superior antibacterial ability compared to PBS and plain Cu-CPNs. Cu-CPNs@EPL at a concentration of 25 μg/mL was found to inhibit the growth of MRSA and PAO1 by 99% (Fig. 6A and B). The bacterial morphology was further analyzed using SEM, which showed surface roughness and cellular deformation in bacteria treated with EPL or Cu-CPNs@EPL (Fig. 6C and D), indicating its strong antibacterial activity. The bacterial colony assay confirmed these results with almost no visible colonies observed in the EPL or Cu-CPNs@EPL groups (Fig. 6E and F).

**Fig. 7:**
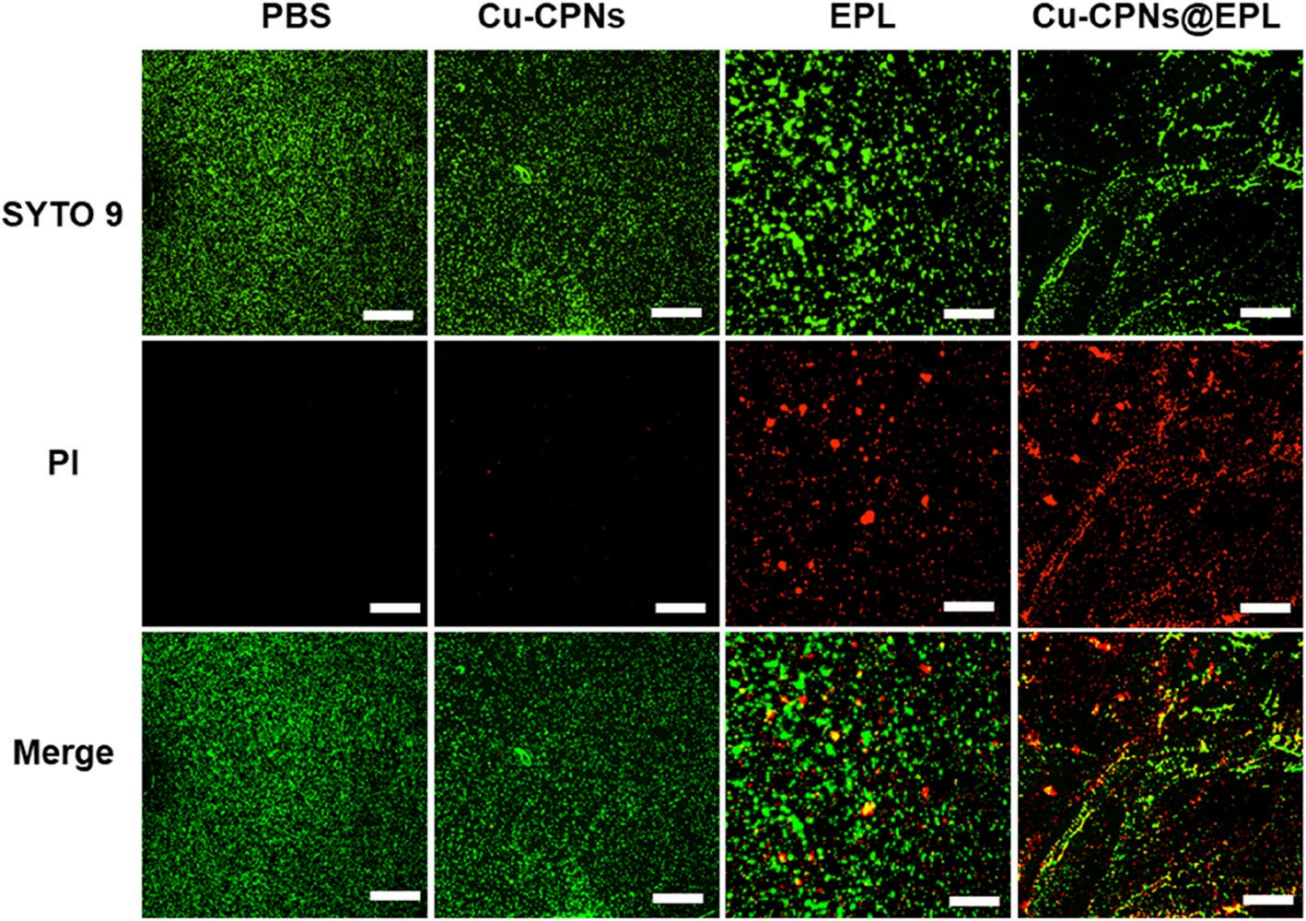
Living/dead bacterium staining of MRSA by SYTO 9/PI after exposure to PBS, Cu-CPNs, EPL, and Cu-CPNs@EPL, respectively. (green fluorescence: SYTO 9 staining, representing live and dead bacteria; red fluorescence: PI staining, representing dead bacteria, scale bar: 100 μm)

The antibacterial efficacy of Cu-CPNs@EPL was evaluated using SYTO 9/PI kit. The SYTO 9 stain was used to stain both live and dead Gram-positive and Gram-negative bacteria with green fluorescence, while dead cells with damaged membranes were stained with red fluorescence dye PI. As seen in Fig. 7 and Fig. S6, while both MRSA and PAO1 remained alive in the PBS and Cu-CPNs groups, most MRSA and PAO1 were dead in the EPL and Cu-CPNs@EPL groups.

### Bacterial metabolomics analysis

**Fig. 8:**
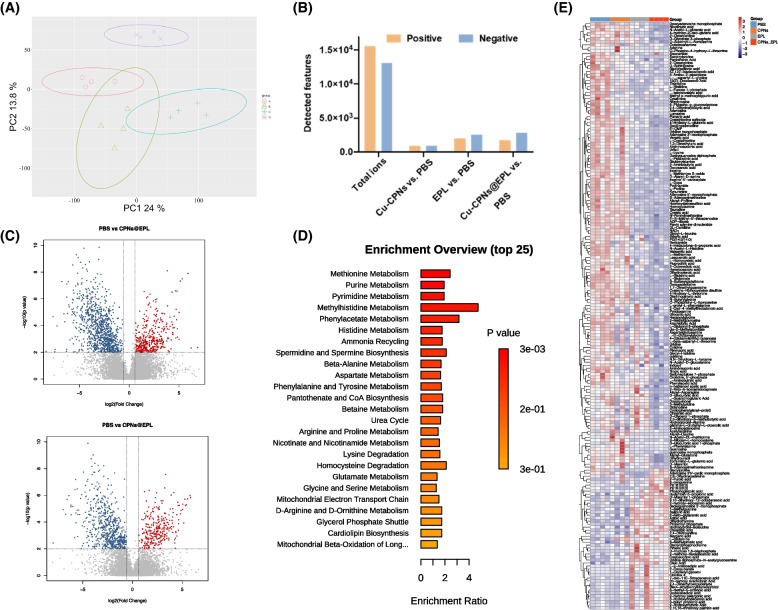
The metabolic analysis of the bacteria in different treatment groups. **A** PCA of PBS, Cu-CPNs, EPL, and Cu-CPNs@EPL treated groups in the positive ion mode; **B** The illustrated volcano plot in comparison of PBS and Cu-CPNs@EPL treatment; **C** The total detected features and the significant difference features in different groups; **D** Enrichment analysis of the control (PBS) group in comparison with Cu-CPNs@EPL-treated group; **E** Heatmap of the significant different metabolites of the bacteria in different treatment groups

The treatment with Cu-CPNs, Cu-CPNs@EPL, and EPL resulted in a significant shift in the bacterial metabolic profile, with the largest change seen in the Cu-CPNs@EPL group (Fig. 8A). This change was reflected in the number of significant difference ions. A total of 15,570 and 13,131 ions were detected in the positive and negative ion modes after various treatments, respectively. The Cu-CPNs, EPL, and Cu-CPNs@EPL-treated groups showed 940, 1,999, and 1,744 significant difference ions (*p* < 0.01, |Fold change|> 1.5), respectively, compared to the PBS group in the positive ion mode, as well as 7,152, 447, and 2,656 significant difference ions (*p* < 0.01, |Fold change|> 1.5), respectively, compared to the PBS group in the negative ion mode (Fig. 8B and C). A total of 202 metabolites were identified, and it was observed that the methionine metabolism, purine metabolism, pyrimidine metabolism, and methylhistidine metabolism pathways were highly affected after Cu-CPNs@EPL treatment, as shown in Fig. 8D. In addition, the enrichment analysis of the Cu-CPNs-treated group in Fig. S7A indicated that Cu-CPNs treatment mainly affected histidine metabolism, nitrogen metabolism, D-glutamine and D-gluamate metabolism. On the other hand, EPL treatment mainly affected purine metabolism, arginine biosynthesis, cysteine and methionine metabolism, and pyrimidine metabolism, as shown in Fig. S7B.

These metabolites showed a decreasing trend in these pathways (Fig. 8E). Methionine is an essential amino acid and is also a key component of S-adenosyl methionine (SAM) [37], a cellular carrier of methyl groups involved in cell growth, repair, and maintenance of the cell membrane’s phospholipid layer. Interestingly, SAM was presented as 0.26 fold change after the Cu-CPNs@EPL treatment (*p* = 0.037), indicating that the bacteria suffered from the lack of methylation, which affected the reproduction rate of bacteria. Besides, we also observed other important pathways that affect bacterial reproduction, i.e., purine and pyrimidine metabolisms, which are closely related to DNA replication and serve as main energy carriers, were down-regulated in the Cu-CPNs@EPL treated group. Furthermore, the subunits of nucleic acids and precursors for the synthesis of nucleotide cofactors, such as nicotinamide adenine dinucleotide (NAD) and SAM [38, 39], were also reduced in Cu-CPNs@EPL group.

### In vivo MRSA-infected wound healing evaluation

**Fig. 9:**
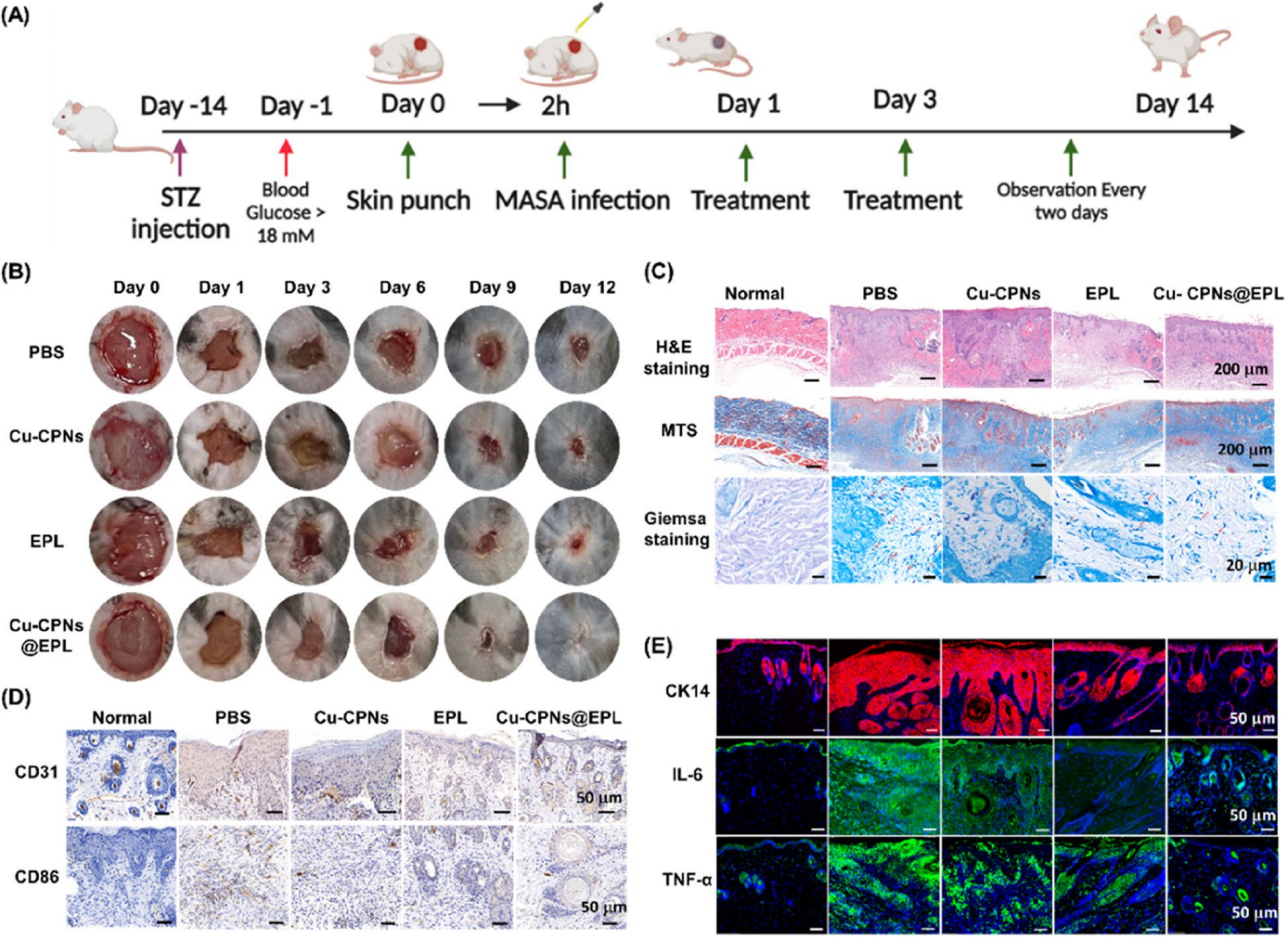
**A** Schematic diagram of in vivo anti-infective efficiency of different treatment on the diabetic mice wound infected by MRSA. **B** Representative pictures of MRSA-infected full-thickness wounds on C57BL/6 mice with different treatments on days 0, 1, 3, 6, 9, and 12. **C** Representative histopathological observation of H&E staining, MTS, and Giemsa staining of dermal wound paraffin section collected on day 12. **D** Immunohistochemical staining of CD31 and CD86 of the dermal wound paraffin section collected on day 12. **E** Immunofluorescence staining of CK14 (red), IL-6 (green), TNF-α (green) and cell nucleus (blue) of dermal wound paraffin section collected on day 12

The efficacy of Cu-CPNs@EPL on wound healing and antibacterial activity was evaluated on a diabetic mouse model infected with MRSA. The results showed that the wounds of the mice treated with PBS, Cu-CPNs, and EPL exhibited significant inflammation, but the treatment with Cu-CPNs@EPL greatly accelerated wound healing (Fig. 9B). After 12 days of treatment, the wound tissues were harvested for analysis. The results of both H&E staining indicated better wound healing in the Cu-CPNs@EPL-treated group compared to the control groups. The collagen fiber staining with MTS in Fig. 9C showed that collagen deposition (blue staining) was obviously increased in the Cu-CPNs@EPL-treated group for infected wounds, indicating that the wounds were healing and closed. The Giemsa staining showed a decrease in the number of bacteria in the wound tissue of the EPL and Cu-CPNs@EPL group, indicating the effectiveness of Cu-CPNs@EPL in treating infected wounds.

The immunohistochemistry staining of CD31 and CD86 was performed to evaluate angiogenesis and pro-inflammatory M1 macrophages, respectively. Results showed that the expression of CD31 in the Cu-CPNs@EPL group was higher than in the control and EPL groups at day 12 (Fig. 9D), indicating an accelerating effect on angiogenesis due to the presence of copper ions [40, 41]. The expression of CD86 in Cu-CPNs@EPL group was significantly lower than that in control group, indicating the anti-inflammatory effect of Cu-CPNs@EPL in chronic wound healing. In addition, the renascent epithelial thickness was stained by CK 14 antibody in different groups for quantitative assessments, and the normal epithelial tissues were also harvested as the negative control (Fig. 9E). The epithelial thickness was also found to be thinner in the EPL and Cu-CPNs@EPL groups compared to the PBS group, but similar to normal tissue in the Cu-CPNs@EPL group, indicating that the Cu-CPNs@EPL could accelerate wound recovery. Furthermore, the expression of pro-inflammatory cytokines TNF-α and IL-6 was reduced in the Cu-CPNs@EPL group (Fig. 8E), indicating M1-to-M2 polarization of macrophages, which is beneficial for wound closure. The H&E staining of major organs also showed excellent histocompatibility (Fig. S8) and in vivo biosafety of Cu-CPNs@EPL.

## Discussion

Diabetic ulceration is a common complication that can lead to chronic wound infections, hindering the healing process and negatively impactthe quality of life. To treat infected wounds, various therapeutic modalities including peptides, therapeutic antimicrobials, and stem cell therapy have been developed [7, 42]. In addition, a variety of antibacterial nanomaterials such as gold nanoparticles, copper oxide, and two-dimensional nanohybrid, nanofibrous dressing, and hydrogels have emerged as alternatives to antibiotics [8, 43–47]. However, these nanomaterials do not always effectively control chronic wound infections due to their limited functions and potential cytocompatibility issues, which can impact their biosafety and applications in treating diabetes-related chronic wound infections. In the present study, the antioxidant nanozyme was designed using GMP as the coordination scaffold and copper ion as the center of CPNs to reduce ROS levels and enhance wound healing. Copper ions play a crucial role in cellular redox reactions and are vital to keep in the Cu(I) valence state for optimal biomedical use. Copper-based nanomaterials with Cu(I) state component are essential for scavenging endogenous ROS and have a strong impact on wound healing through contributions to angiogenesis and collagen deposition. Nucleotides are regarded as metal ligands due to their ability to coordinate with transition metal ions. The non-covalent interactions between GMP and copper ions induce the formation of responsive and amorphous Cu-CPNs. Previous studies have suggested that the nitrogen and oxygen atoms of nucleobases and phosphate groups in GMP, owing to the presence of lone pair electrons, can act as potential binding sites for metal ions [48, 49]. The antioxidant properties of GMP allowed the Cu-CPNs to retain the Cu(I) valence state, resulting in reductive activity and the ability to mimic antioxidant enzymes. To enhance the anti-infection capacity, an antibacterial component, EPL, was integrated to the Cu-CPNs to produce the Cu-CPNs@EPL complex through a one-step self-assembly process, which showed strong electropositivity and the ability to interact with bacteria for potent antibacterial effects.

Cu-CPNs and Cu-CPNs@EPL showed the effective SOD-mimicking activity according to the measurement of the O_2_^•−^ scavenging efficiency ininhibiting the formazan formation. Using methionine as this electron donor, riboflavin reacts with oxygen to produce O_2_^•−^ in the presence of oxygen and light, allowing the photo-excited reduction of riboflavin. The O_2_^•−^ reduces the slightly yellow NBT to blue formazan, and SOD inhibits the formation of blue formazan by catalysing the O_2_^•−^ disproportionation reaction to produce O_2_ with H_2_O_2_. The Cu-CPNs and Cu-CPNs@EPL were also confirmed to exhibit excellent GPx activity in scavenging H_2_O_2_ through enzymatic substrate reaction validation. Moreover, to confirm the GPx-mimicking activity of Cu-CPNs under physiological conditions, the metabolites extracted from mouse liver tissue were treated with Cu-CPNs and PBS, respectively. The results demonstrate the effective antioxidase-mimicking activity and ROS-scavenging abilities of Cu-CPNs and Cu-CPNs@EPL.

To evaluate the antibacterial efficacy of Cu-CPNs@EPL, the material was tested against Gram-positive MRSA and Gram-negative PAO1 bacteria. The cationic charge of EPL and Cu-CPNs@EPL caused them to strongly adhere to the negatively charged bacterial cell membranes, breaking the transmembrane potential and osmotic balance and disrupting membrane fusion, leading to bacteria clustering. These results suggest that Cu-CPNs@EPL is a potent agent that effectively kills bacteria with serious damage to the cell membrane. The resulting Cu-CPNs@EPL showed high inhibition efficiency against MRSA and PAO1.

To uncover the mechanisms of action of the prepared nanomaterials against bacteria, a metabolomic analysis was performed on MRSA, bacterial metabolomics analysis indicated that the Cu-CPNs@EPL primarily impacted the integrity of the bacterial cell membrane, causing death of bacteria. Furthermore, these disturbed metabolic findings indicated that the Cu-CPNs@EPL might mainly impact the integrity of bacterial membrane, inhibit the synthesis of nucleic acids, both of which could induce subsequent bacterial death. Our study showed that Cu-CPNs@EPL has both antioxidant nanozyme properties and antibacterial properties, as demonstrated by its ability to inhibit bacterial growth in vitro.

In an animal study with a MRSA infected diabetic wound model, the Cu-CPNs@EPL treatment effectively eradicated MRSA infection, relieved oxidative stress, and induced angiogenesis, creating a favorable microenvironment for inflammation reduction, cell proliferation, vascularization, tissue formation at the wound site. Overall, Cu-CPNs@EPL showed a promising therapeutic effect for treating MRSA-infected diabetic cutaneous wounds with good biosafety.

## Conclusion

In this study, we developed a type of Cu-CPNs nanozyme with excellent GPx and SOD-like activity that mimics an antioxidant defense system. To enhance the antibacterial and antioxidant properties, the nanozyme was combined with a typical cationic and antibacterial EPL polymer through a one-pot self-assembly process. The resulting Cu-CPNs@EPL showed potent antibacterial activity against MRSA and PAO1, as well as strong ROS scavenging ability. The efficacy of Cu-CPNs@EPL was evaluated in a diabetic mouse model with MRSA-infected skin wounds and showed satisfactory antibacterial and anti-inflammatory performance, along with excellent biocompatibility. These results suggest that Cu-CPNs@EPL is a promising multifunctional antioxidant nanozyme-based therapeutic for the treatment of drug-resistant bacteria-infected wounds.

### Supplementary Information

**Additional file 1: Figure S1.** The coordination form in the structural unit of the as-prepared Cu-CPNs. **Figure S2.** (A) Powder XRD patterns GMP, Cu-CPNs, EPL, and Cu-CPNs@EPL, respectively. **Figure S3.** N_2_ adsorption-desorption isotherms of (A) Cu-CPNs and (C) Cu-CPNs@EPL. The corresponding pore-size distribution curve of (B) Cu-CPNs, and (D) Cu-CPNs@EPL. Before N2 adsorption-desorption, the sample was freeze-dried and degassed at room temperature for 10 h. **Figure S4.** CCk-8 assay of (A) Raw 264.7 and (B) NIH 3T3 cells under different concentrations of Cu-CPNs and Cu-CPNs@EPL. The Live/Dead staining of Cu-CPNs and Cu-CPNs@EPL treated (C) Raw 264.7 and (D) NIH 3T3 cells (green fluorescence, Calcein AM indicates live cells; red fluorescence: propidium iodide indicates dead cells, Scale bar: 100 μm). **Figure S5.** Representative ROS staining (green fluorescence) of NIH 3T3 cells under different concentration of Cu-CPNs-treatment (Scale bar: 100 μm). **Figure S6.** Living/dead bacterium staining of PAO1 by SYTO 9/PI after exposure to PBS, Cu-CPNs, EPL, and Cu-CPNs@EPL, respectively. (green fluorescence: SYTO 9 staining, representing live and dead bacteria; red fluorescence: PI staining, representing dead bacteria, scale bar: 100 μm). **Figure S7.** Enrichment analysis of the control (PBS) group in comparison with (A) Cu-CPNs-treated group and (B) EPL-treated group. **Figure S8.** H&E staining of major organs slices including heart, liver, spleen, lung, and kidney of mice in normal, PBS, Cu-CPNs, EPL, and Cu-CPNs@EPL treatment group. (Scale bar: 50 μm).

## Data Availability

For data and materials requests, please contact the authors.

## Abbreviations

ROS: Reactive oxygen species
Cu-CPNs: Copper-coordination polymer nanoparticles
EPL: ε-Polylysine
CPNs: Coordination polymer nanoparticles
GMP: Guanosine monophosphate
MRSA: Methicillin-resistant Staphylococcus aureus
PAO1: Pseudomonas aeruginosa
DMEM: Dulbecco’s modified Eagle’s medium
PI: Propidium iodide
GSSG: Oxidized glutathione
GPx: Glutathione peroxidase
SOD: Superoxide dismutase
SAM: S-adenosyl methionine
NAD: Nicotinamide adenine dinucleotide
NBT: Nitrotetrazolium Blue chloride
SEM: Scanning electron microscopy
H&E: Hematoxylin and eosin
MTS: Masson’s trichrome staining
LC–MS/MS: Liquid chromatography-tandem mass spectrometry
PCA: Principal component analysis
STZ: Streptozotocin
CK 14: Cytokeratin 14
