# Supplementary material for: Multifunctional nanozyme-reinforced copper-coordination polymer nanoparticles for drug-resistance bacteria extinction and diabetic wound healing
Source: Biomater Res. 2023 Sep 18;27:88. doi: 10.1186/s40824-023-00429-z (PMC10506277; doi:10.1186/s40824-023-00429-z)
Supplement: Supplementary file 1 — Additional file 1: Figure S1. The coordination form in the structural unit of the as-prepared Cu-CPNs. Figure S2. (A) Powder XRD patterns GMP, Cu-CPNs, EPL, and Cu-CPNs@EPL, respectively. Figure S3. N2 adsorption-desorption isotherms of (A) Cu-CPNs and (C) Cu-CPNs@EPL. The corresponding pore-size distribution curve of (B) Cu-CPNs, and (D) Cu-CPNs@EPL. Before N2 adsorption-desorption, the sample was freeze-dried and degassed at room temperature for 10 h. Figure S4. CCk-8 assay of (A) Raw 264.7 and (B) NIH 3T3 cells under different concentrations of Cu-CPNs and Cu-CPNs@EPL. The Live/Dead staining of Cu-CPNs and Cu-CPNs@EPL treated (C) Raw 264.7 and (D) NIH 3T3 cells (green fluorescence, Calcein AM indicates live cells; red fluorescence: propidium iodide indicates dead cells, Scale bar: 100 μm). Figure S5. Representative ROS staining (green fluorescence) of NIH 3T3 cells under different concentration of Cu-CPNs-treatment (Scale bar: 100 μm). Figure S6. Living/dead bacterium staining of PAO1 by SYTO 9/PI after exposure to PBS, Cu-CPNs, EPL, and Cu-CPNs@EPL, respectively. (green fluorescence: SYTO 9 staining, representing live and dead bacteria; red fluorescence: PI staining, representing dead bacteria, scale bar: 100 μm). Figure S7. Enrichment analysis of the control (PBS) group in comparison with (A) Cu-CPNs-treated group and (B) EPL-treated group. Figure S8. H&E staining of major organs slices including heart, liver, spleen, lung, and kidney of mice in normal, PBS, Cu-CPNs, EPL, and Cu-CPNs@EPL treatment group. (Scale bar: 50 μm). [file 40824_2023_429_MOESM1_ESM.docx]

**Supporting Information**

Multifunctional Antioxidant Nanozyme-Reinforced Copper-Coordination Polymer Nanoparticles for Drug-Resistance Bacteria Extinction and Diabetic Wound Therapy

Jiahui Zhao ^a, b, #^, Tengfei Xu ^a, c, #^, Jichao Sun ^a^, Haitao Yuan ^a, b^, Mengyun Hou ^a^, Zhijie Li ^a, *^, Jigang Wang ^a, *^, Zhen liang ^a, *^

^a^Department of Geriatrics and Shenzhen Clinical Research Centre for Geriatrics, Shenzhen People’s Hospital (The Second Clinical Medical College, Jinan University, The First Affiliated Hospital, Southern University of Science and Technology), Shenzhen, Guangdong 518020, PR China

^b^Integrated Chinese and Western Medicine Postdoctoral Research Station, Jinan University, Guangzhou 510632, P.R. China.

^c^College of Pharmaceutical Sciences, Zhejiang University, Hangzhou 310058, P.R. China.

^#^Jiahui Zhao and Tengfei Xu contributed equally to this work.

*Corresponding authors:

Jigang Wang, email: wangjigang@u.nus.edu

Zhen Liang, email: Liang.zhen@szhospital.com

Zhijie Li, email: [li.zhijie@szhospital.com](mailto:li.zhijie@szhospital.com)


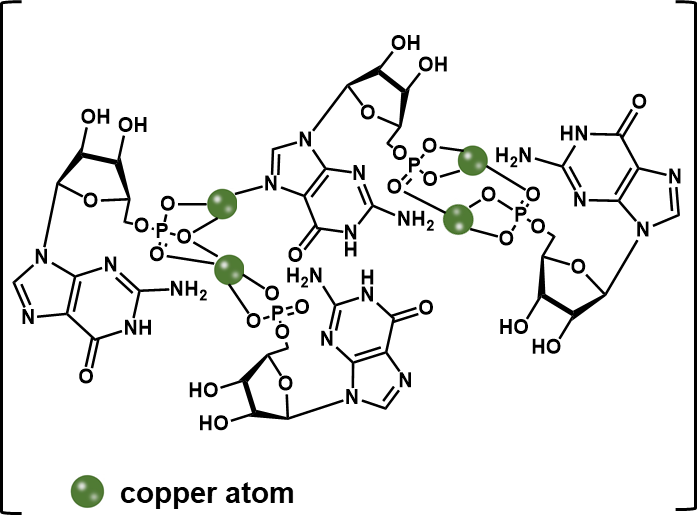


Figure S1. The coordination form in the structural unit of the as-prepared Cu-CPNs.


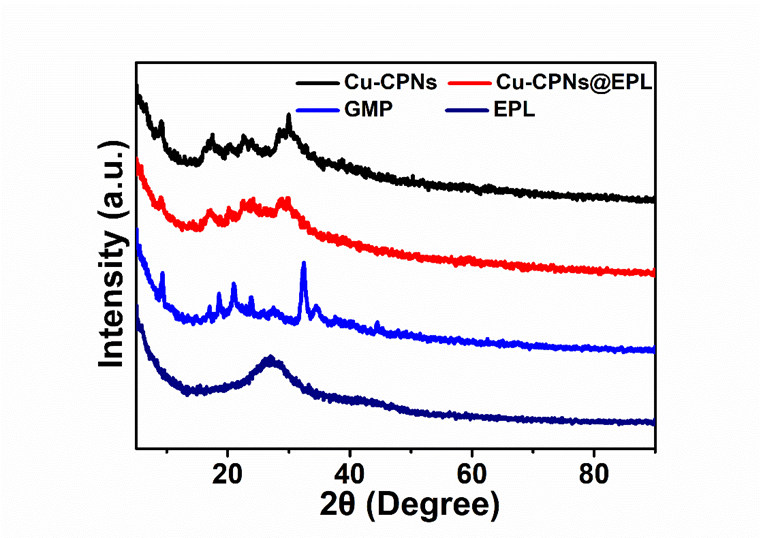


Figure S2. (A) Powder XRD patterns GMP, Cu-CPNs, EPL, and Cu-CPNs@EPL, respectively.


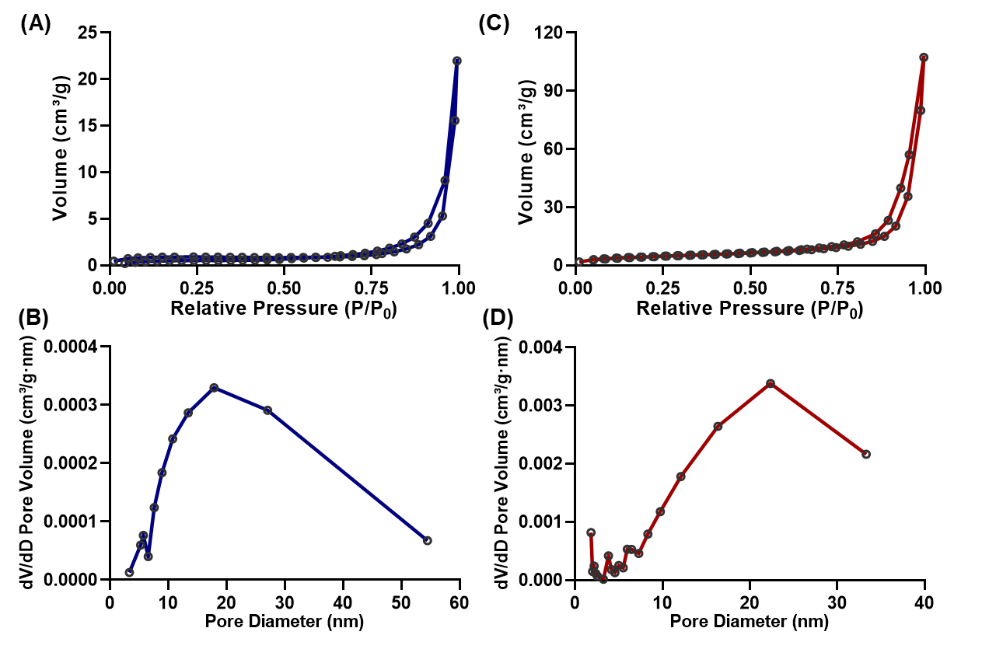


Figure S3. N_2_ adsorption-desorption isotherms of (A) Cu-CPNs and (C) Cu-CPNs@EPL. The corresponding pore-size distribution curve of (B) Cu-CPNs, and (D) Cu-CPNs@EPL. Before N_2_ adsorption-desorption, the sample was freeze-dried and degassed at room temperature for 10 h.


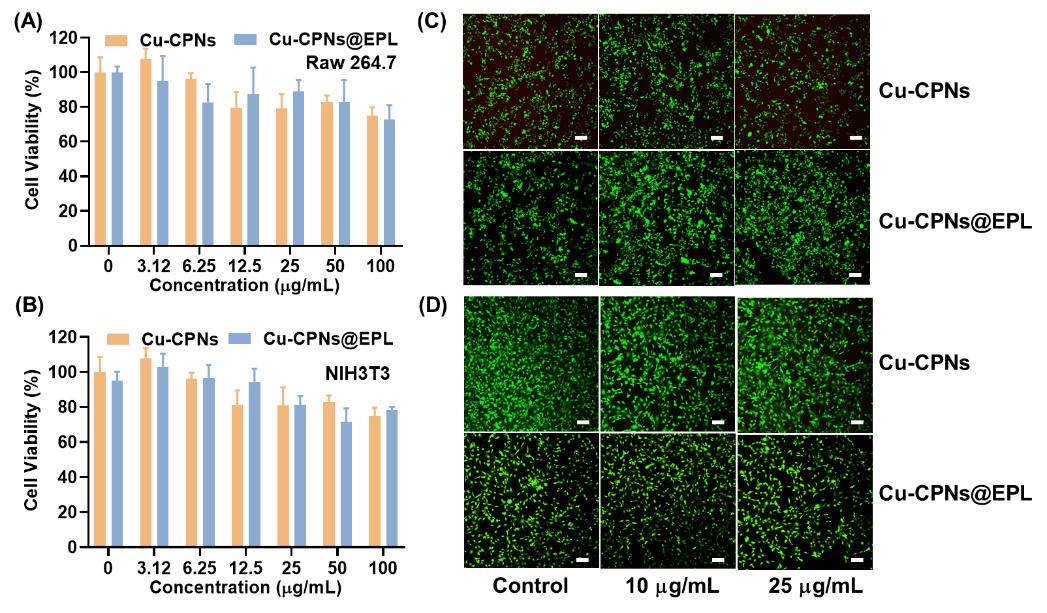


Figure S4. CCk-8 assay of (A) Raw 264.7 and (B) NIH 3T3 cells under different concentrations of Cu-CPNs and Cu-CPNs@EPL. The Live/Dead staining of Cu-CPNs and Cu-CPNs@EPL treated (C) Raw 264.7 and (D) NIH 3T3 cells (green fluorescence, Calcein AM indicates live cells; red fluorescence: propidium iodide indicates dead cells, Scale bar: 100 μm)


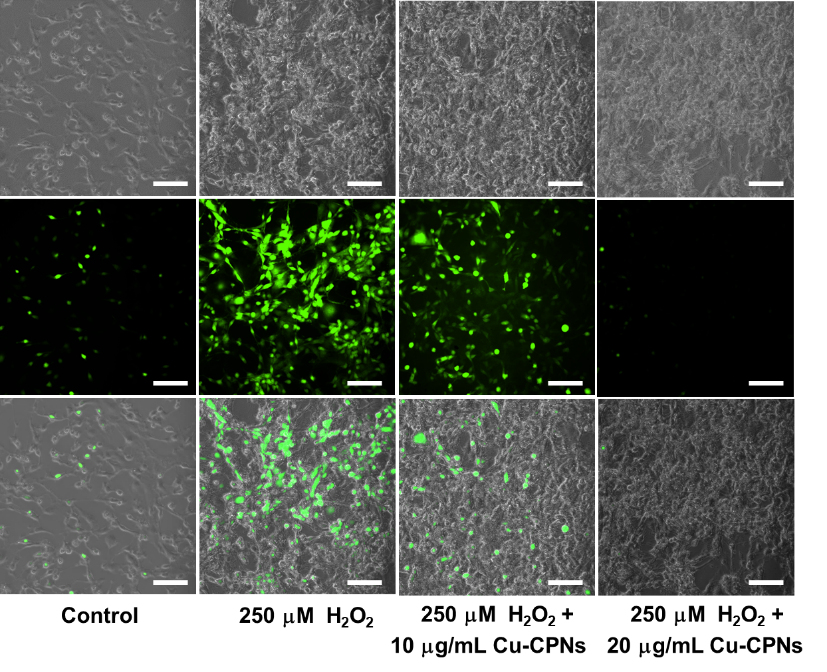


Figure S5. Representative ROS staining (green fluorescence) of NIH 3T3 cells under different concentration of Cu-CPNs-treatment (Scale bar: 100 μm).


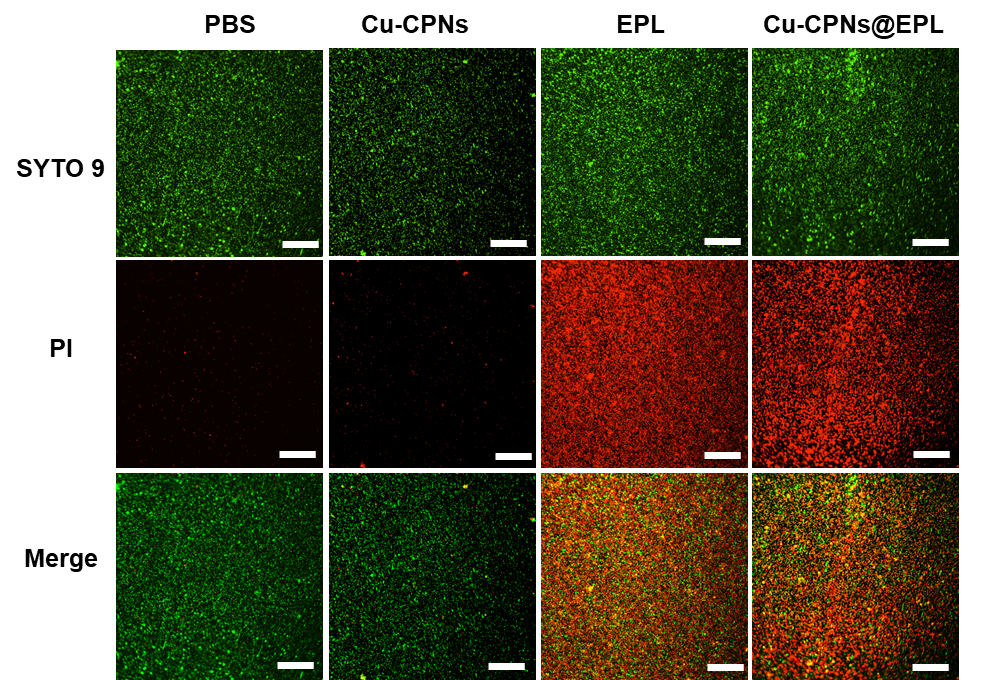


Figure S6. Living/dead bacterium staining of PAO1 by SYTO 9/PI after exposure to PBS, Cu-CPNs, EPL, and Cu-CPNs@EPL, respectively. (green fluorescence: SYTO 9 staining, representing live and dead bacteria; red fluorescence: PI staining, representing dead bacteria, scale bar: 100 μm)


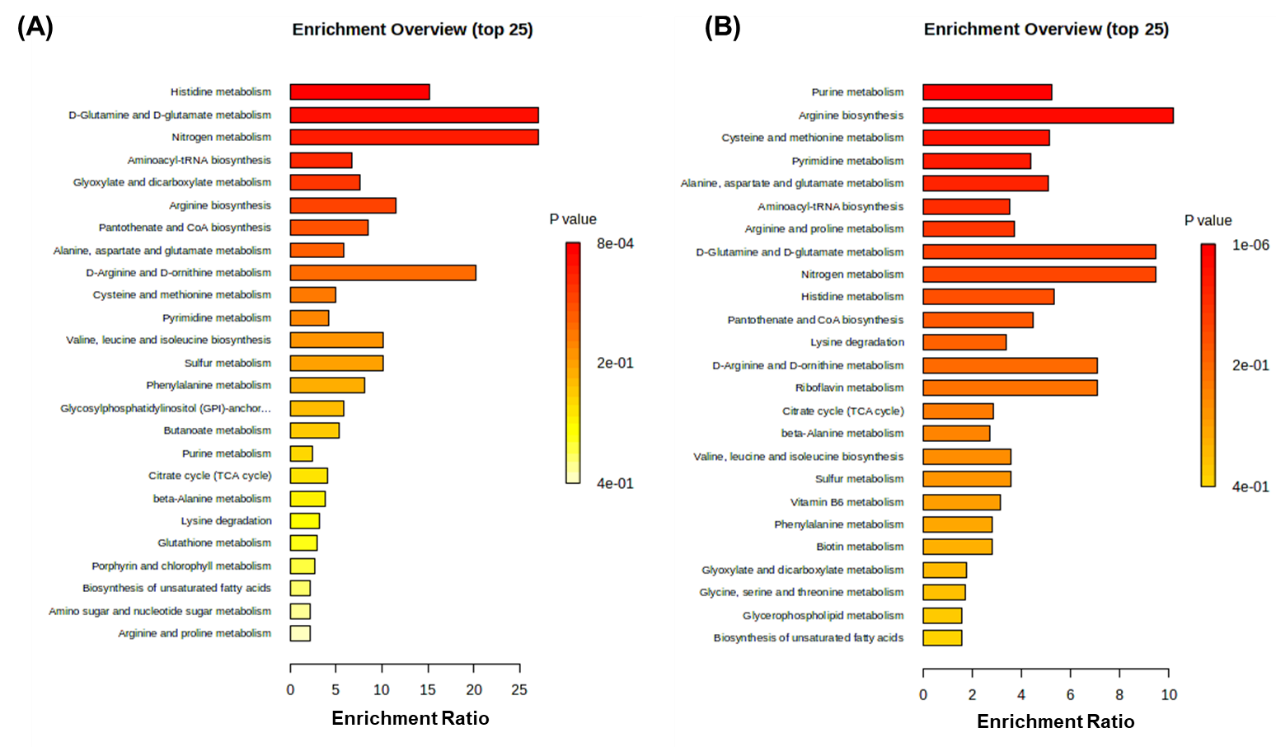


Figure S7. Enrichment analysis of the control (PBS) group in comparison with (A) Cu-CPNs-treated group and (B) EPL-treated group.


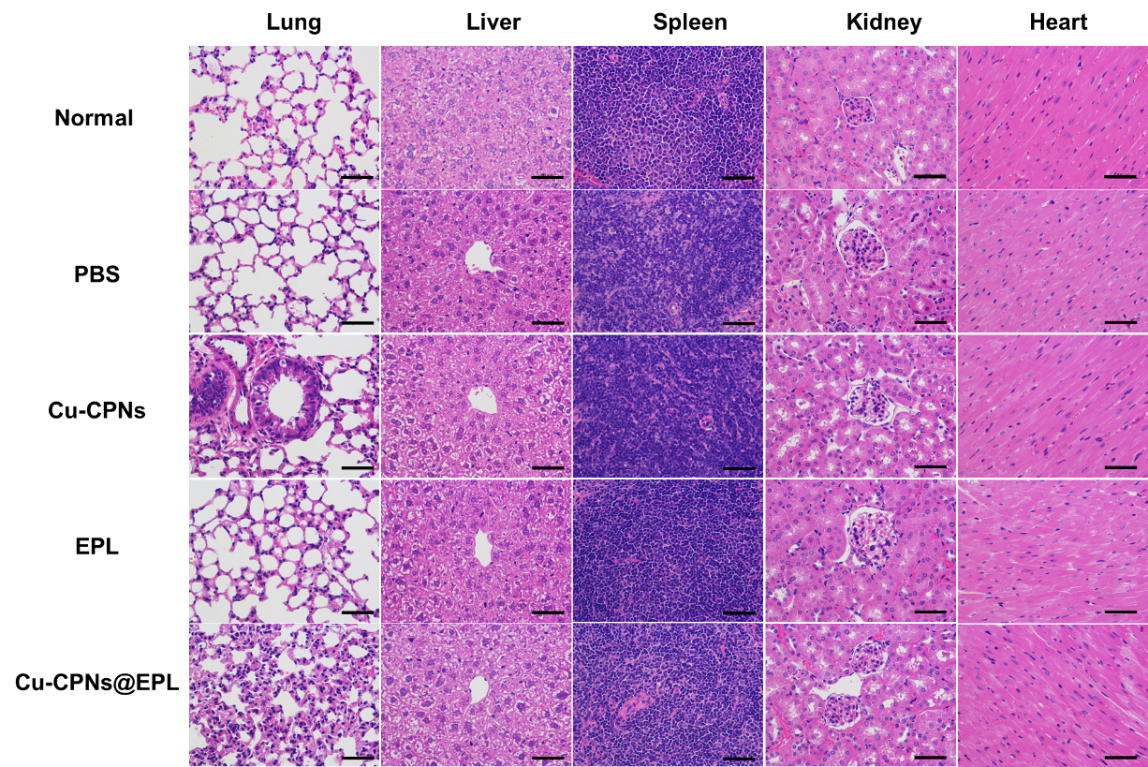


Figure S8. H&E staining of major organs slices including heart, liver, spleen, lung, and kidney of mice in normal, PBS, Cu-CPNs, EPL, and Cu-CPNs@EPL treatment group. (Scale bar: 50 μm)
